# Comparative Evaluation of UV-Vis Spectroscopy-Based Approaches for Hemoglobin Quantification: Method Selection and Practical Insights

**DOI:** 10.3390/biom14091046

**Published:** 2024-08-23

**Authors:** Clara Coll-Satue, Michelle Maria Theresia Jansman, Leticia Hosta-Rigau

**Affiliations:** Center for Nanomedicine and Theranostics, Department of Health Technology, Technical University of Denmark, Nils Koppels Allé, Building 423, 2800 Kongens Lyngby, Denmark

**Keywords:** protein quantification, absorbance-based assays, hemoglobin, blood substitutes, hemoglobin-based oxygen carriers

## Abstract

The growing demand for effective alternatives to red blood cells (RBCs) has spurred significant research into hemoglobin (Hb)-based oxygen carriers (HBOCs). Accurate characterization of HBOCs—including Hb content, encapsulation efficiency, and yield—is crucial for ensuring effective oxygen delivery, economic viability, and the prevention of adverse effects caused by free Hb. However, the choice of quantification methods for HBOCs is often driven more by tradition than by a thorough assessment of available options. This study meticulously compares various UV-vis spectroscopy-based methods for Hb quantification, focusing on their efficacy in measuring Hb extracted from bovine RBCs across different concentration levels. The findings identify the sodium lauryl sulfate Hb method as the preferred choice due to its specificity, ease of use, cost-effectiveness, and safety, particularly when compared to cyanmethemoglobin-based methods. Additionally, the study discusses the suitability of these methods for HBOC characterization, emphasizing the importance of considering carrier components and potential interferences by analyzing the absorbance spectrum before selecting a method. Overall, this study provides valuable insights into the selection of accurate and reliable Hb quantification methods, which are essential for rigorous HBOC characterization and advancements in medical research.

## 1. Introduction

While blood transfusion remains a crucial, safe, and common procedure for saving lives, it is not without limitations and risks, such as the need for blood group compatibility screening and the detection of infectious agents [1]. Furthermore, the short shelf life of blood products and their limited availability exacerbate the current blood shortage—a situation expected to worsen with the demographic shifts of an aging and growing population [2]. Therefore, the development of red blood cell (RBC) substitutes is a pressing need in biomedicine. 

Since hemoglobin (Hb) is the main component of RBCs and the molecule responsible for oxygen transport, a large amount of research has focused on developing Hb-based oxygen carriers (HBOCs) [3]. These semi-synthetic systems, extensively studied in the last three decades, aim to address several key limitations of donor blood, including potential shortages, the necessity for cross-matching, infection risks, limited storage life, and specific storage requirements [4]. 

For the successful development of HBOCs, it is essential to employ rigorous and reliable methods to accurately assess the Hb content, encapsulation efficiency (EE), and yield. These parameters are vital for confirming the ability of HBOCs to deliver adequate oxygen once administered into the body and are also economically significant, as yield and EE are key metrics in the production process [5]. Additionally, quantifying potential Hb release from HBOCs is essential because free intravascular Hb can cause severe adverse effects, such as renal toxicity, vasoconstriction, and subsequent cardiovascular complications [6]. An underestimation of the quantity of free Hb released could lead to the oversight of adverse effects potentially resulting in the failure of the HBOC. Conversely, overestimation might raise unfounded concerns or even lead to unnecessarily terminating the development process.

Various methods have been reported to quantify Hb after its extraction from common sources such as bovine or outdated human RBCs and to evaluate the drug loading, EE, and yield of the resulting HBOCs [7,8,9,10,11,12,13]. For instance, Baek et al. used the Coomassie Plus protein assay kit to assess the Hb concentration of different poly-Hbs [14]. Yu et al. calculated the Hb content and EE of biconcave Hb-based microcapsules by measuring the absorbance (Abs) of the Soret peak by ultraviolet–visible (UV-vis) spectroscopy [11]. In another study, Pacheco et al. quantified the Hb content of their silk fibroin HBOCs by the cyanmetHb method [15]. Our group has thus far relied on the bicinchoninic acid assay (BCA) to quantify Hb following extraction from bovine RBCs [16,17,18]. However, for HBOCs characterization, we have employed various methods, including the BCA assay, the Coomassie blue assay, and measuring the Abs of the Soret peak by UV-vis [10,12,19]. It is worth noting that while some of these methods are specific to Hb (e.g., cyanmetHb), others are general protein quantification methods (e.g., the BCA and the Bradford assay). Using non-specific methods without confirming the absence of other proteins could result in inaccurate HBOC characterization.

**Scheme 1:**
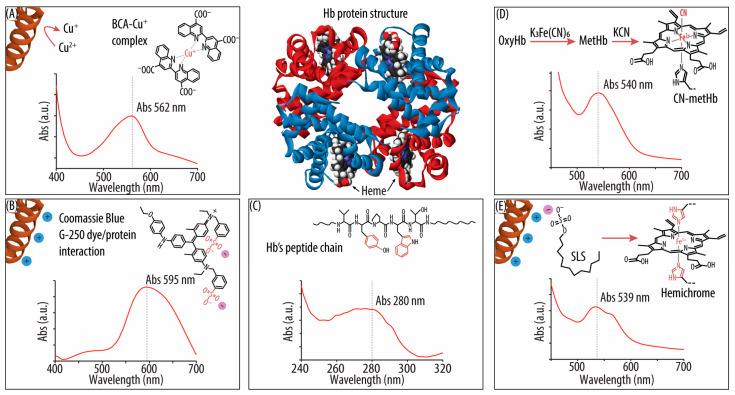
Representation of the different hemoglobin (Hb) quantification methods: (**A**) The bicinchoninic acid (BCA) assay, showing the biuret reaction, the BCA–cuprous ion (Cu^+^) complex formed, and its characteristic absorbance (Abs) spectrum. (**B**) The Bradford Plus assay (Coomassie blue method), showing the interaction of the Coomassie blue G-250 dye and a protein and its characteristic Abs spectrum. (**C**) The method of Abs at 280 nm (Abs280), showing a fragment of the Hb beta chain amino acid (AA) sequence (i.e., residues 34–38, VYPWT), with two representative aromatic AAs that contribute to the Abs signal at 280 nm. The characteristic Abs spectrum in that region is also shown. (**D**) The cyan (CN)–Hb method, which starts with the oxidation of oxygenated Hb (oxyHb) into methemoglobin (metHb) by potassium hexacyanoferrate (III) K_3_[Fe(CN)_6_]. The addition of potassium cyanide (KCN) results in the formation of the CN-metHb complex. The characteristic CN-metHb Abs spectrum is also shown. (**E**) The sodium lauryl sulfate (SLS)–Hb method, showing the interactions between the SLS head group and the positively charged peptide chains of Hb, as well as the hemichrome structure. The Abs spectrum of the SLS-Hb complex is also shown.

Despite the importance of choosing the right Hb quantification method, the decision is often made based on the speed of analysis, availability of reagents, or mere habit [20]. Remarkably, despite the variety of Hb quantification methods available in the literature, a comprehensive comparison of these techniques has not yet been conducted. This study aims to fill this gap by evaluating the accuracy of the most widely used Hb quantification methods in the HBOC field and highlighting the advantages and limitations of each. Our main objective is to provide a comparison of these methods to guide researchers in selecting the most appropriate technique for accurate Hb quantification. We focus on UV-vis spectroscopy-based methods owing to their widespread use, rapidity, and accessibility. To detect potential protein contaminants following extraction from bovine RBCs, both non-specific (i.e., BCA, Coomassie blue (CB), and Abs at 280 nm (Abs_280_)) and Hb-specific (i.e., cyanHb (CN-Hb) and sodium lauryl sulfate (SLS)–Hb)) quantification methods are used to determine the concentrations of three different Hb stocks obtained from bovine RBCs (Scheme 1). Our findings point towards the SLS-Hb method as the preferred one due to its specificity, ease of use, cost efficiency, safety, high accuracy, and precision.

## 2. Materials and Methods

### 2.1. Materials

Tris(hydroxymethyl)aminoethane (TRIS), sodium chloride (NaCl), toluene, potassium cyanide (KCN), potassium hexacyanoferrate (III) (K_3_[Fe(CN)_6_]), SLS, and Hb from bovine blood were purchased from Merck Life Science A/S (Søborg, Denmark). Fresh bovine blood with citrate was purchased from SSI Diagnostica A/S (Hillerød, Denmark). A Pierce^TM^ BCA protein assay kit containing BCA reagent A (i.e., sodium carbonate, sodium bicarbonate, BCA, and sodium tartrate in 0.1 M sodium hydroxide) and BCA reagent B (i.e., 4% cupric sulfate), as well as a Pierce^TM^ Coomassie Plus assay kit containing Pierce Coomassie Plus reagent (i.e., Coomassie G-250 dye, methanol, phosphoric acid, and solubilizing agents in water) were purchased from Thermo Fisher Scientific (Roskilde, Denmark). 

### 2.2. Hb Extraction from Bovine RBCs

Hb was extracted from bovine blood following a reported protocol [21]. The blood was washed three times (2000× *g*, 20 min, 4 °C) with a 0.9% NaCl solution (1:1 volume ratio) using a high-speed centrifuge (SL16R centrifuge, Thermo Fisher Scientific, Hvidovre, Denmark). The resulting pellet containing the RBCs was thoroughly mixed with distilled water and toluene (1:1:0.4 volume ratio). The mixture was placed in a separation funnel and stored overnight at 4 °C. After storage, three distinct layers could be observed, of which the lowest one was a stroma-free Hb solution. That solution was collected, centrifuged (8000× *g*, 20 min, 4 °C), and filtered. Then, 2 mL aliquots were prepared and stored at −80 °C for future use. 

Serial dilutions were performed for the three independent Hb stocks (Hb extracted from the blood of three different cows on different dates) that were known to have a low, a medium, and a high Hb concentration, respectively.

### 2.3. Protein Quantification Methods

**Scheme 2:**
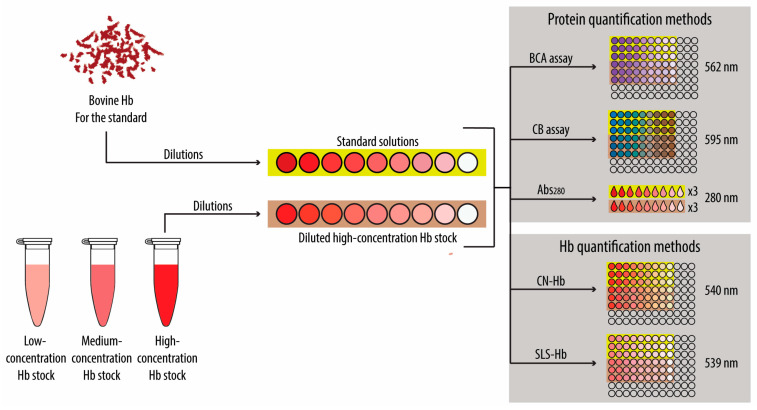
Schematic illustration of the experimental setup for the quantification of hemoglobin (Hb). For simplicity, only the high-concentration Hb stock is shown, but the same process was repeated for the low- and medium-concentration Hb stocks. Briefly, different Hb standards and dilutions of the Hb stocks were prepared. Then, both non-specific (i.e., bicinchoninic acid (BCA) assay, Coomassie blue (CB), and absorbance (Abs) at 280 nm (Abs280)) and Hb-specific quantification methods (i.e., cyan–Hb (CN-Hb) and sodium lauryl sulfate (SLS)–Hb) were conducted by measuring the Abs signal at the specified wavelength for each method.

Lyophilized bovine Hb purchased from Merck Life Science was used to prepare the Hb standards (Scheme 2). Specifically, Hb standards in the concentration ranges of 0–1.5, 0–1, and 0–2 mg mL^−1^ rendered adequate Abs windows for the standard curves using the BCA, CB, and Abs_280_ methods, respectively. Specific dilution ranges for each unknown Hb stock (i.e., low, medium, and high Hb concentration) were chosen to obtain Abs values compatible with the Hb standards. At least six serial dilutions were conducted, resulting in dilution ranges of 25–400× for the low-concentration, 25–600× for the medium-concentration, and 25–700× for the high-concentration Hb stock. All the dilutions were prepared using MQ, which was also used to subtract the background Abs. Detailed information about how the different dilutions were prepared can be found in Tables S1–S4 (Supporting Information). To minimize any handling errors, enough volume of the different solutions was prepared to allow quantification with the three different methods.

#### 2.3.1. BCA Assay

The BCA assay was carried out following the manufacturer instructions for the microplate procedure [22]. Specifically, 25 µL of each Hb standard or Hb stock dilutions were loaded per well in triplicate in transparent 96-well plates (Nunclon^TM^ Delta Surface, Thermo Fisher Scientific, Roskilde, Denmark). Next, 200 µL of BCA working reagent (50:1, reagent A:reagent B) was added to each well. To allow for proper mixing, the 96-well plate was placed in a plate shaker for 30 s covered with aluminum foil. After 30 min of incubation at 37 °C, the Abs spectra in the wavelength range of 400–700 nm were recorded by measuring the Abs signals at 562 nm using a plate reader (Tecan Spark, Tecan Group Ltd., Männedorf, Switzerland). 

#### 2.3.2. CB Assay

The CB assay (also known as the Bradford assay) was carried out following the manufacturer instructions for the microplate procedure [23]. Specifically, 10 µL of each Hb standard or Hb stock dilutions were loaded per well in triplicate using transparent 96-well plate (Nunclon^TM^ Delta Surface, Thermo Fisher Scientific, Roskilde, Denmark). Next, 300 µL of the Coomassie plus reagent, which had been kept outside the refrigerator (i.e., at room temperature) for at least 30 min, was added to each well. To allow for proper mixing, the 96-well plate was placed on a plate shaker, covered with aluminum foil, and incubated for 10 min at room temperature. After incubation, the Abs spectra in the wavelength range of 400–700 nm were recorded measuring the Abs signals at 595 nm using the plate reader.

#### 2.3.3. Abs_280_ Method

First, 3 µL droplets of each Hb standard or Hb stock dilutions were deposited onto the Nanodrop’s pedestal (Nanodrop 2000c, Thermo Fisher Scientific, Waltham, MA, USA). Then, the Abs spectra in the wavelength range of 240–320 nm were recorded measuring the Abs_280_. Each solution was measured three times.

### 2.4. Hb-Specific Quantification Methods 

Lyophilized bovine Hb purchased from Merck Life Science was used to prepare the Hb standards. Specifically, Hb standards in the concentration ranges of 0–20 and 0–5 mg mL^−1^ rendered adequate Abs windows for the standard curves using the SLS-Hb and the CN-Hb method, respectively. Specific dilution ranges for each unknown Hb stock (i.e., low, medium, and high Hb concentrations) were chosen to obtain Abs values compatible with the Hb standards. At least six serial dilutions were conducted, resulting in dilution ranges of 4–100× for the low-concentration, 8–300× for the medium-concentration, and 10–300× for the high-concentration Hb stock. All the dilutions were prepared using TRIS buffer, which was also used to subtract the background Abs. Detailed information on how the different dilutions were prepared can be found in Tables S5–S7 (Supporting information). To minimize any handling errors, enough volume of the different solutions was prepared to allow quantification with the two different methods. 

#### 2.4.1. CN-Hb Method

The CN-Hb method is based on a reported protocol that combines Drabkin’s method and Evelyn–Malloy’s method [24]. Specifically, 135 µL of each Hb standard or Hb stock dilutions were loaded per well in triplicate using transparent 96-well plates (Nunclon^TM^ Delta Surface, Thermo Fisher Scientific, Roskilde, Denmark). Next, 10 µL of K_3_[Fe(CN)_6_] (3% in TRIS buffer) was added to each well followed by the addition of 10 µL of KCN (3% in TRIS buffer). Then, the plate was covered with aluminum foil and placed on the plate shaker for 10 min. After incubation, the Abs spectra in the wavelength range of 450–700 nm were recorded measuring the Abs signals at 540 nm using the plate reader. A magnetic pad (lid lifter) was attached to the lid of the 96-well plate, which made it possible to remove the lid automatically inside the plate reader during measurements, thereby minimizing the exposure to cyanides. 

#### 2.4.2. SLS-Hb Method

The SLS-Hb method is based on a reported protocol [25]. Specifically, 10 µL of each Hb standard or Hb stock dilutions were loaded per well in triplicate using transparent 96-well plates (Nunclon^TM^ Delta Surface, Thermo Fisher Scientific, Roskilde, Denmark). Then, 200 µL of SLS (0.6 mg mL^−1^ in MQ) was added to each well. The plate was covered with aluminum foil and placed on the plate shaker for 5 min at room temperature. After incubation, the Abs spectra in the wavelength range of 450–700 nm were recorded measuring the Abs signals at 539 nm using the plate reader. 

### 2.5. Data Acquisition

The technical triplicates of the blanks (MQ or TRIS buffer) were averaged and subtracted from the Abs values of the Hb standards or Hb stock dilutions. Using Excel software (version 2407, build 16.0.17830.20056), the average Abs of each Hb standard was plotted against its known concentration (mg mL^−1^) in a scatter chart. Then, linear fitting was used, and the equation’s slope, intercept, and R^2^-value were calculated. Finally, the obtained equations were used to determine the concentrations of the three Hb stocks. 

The working range where each method was nearly linear was determined (i.e., 0–1.5 mg mL^−1^ Hb for the BCA assay, 0–1 mg mL^−1^ Hb for the CB assay, 0–5 mg mL^−1^ Hb for the CN-Hb assay, 0–20 mg mL^−1^ Hb for the SLS-Hb assay, and 0–10 mg mL^−1^ Hb for the Abs_280_ method). Although quadratic polynomial fitting could provide more accurate results, linear fitting was chosen for all the methods due to being widely used, more accessible, and easier to work with.

### 2.6. Statistical Analysis

Statistical analysis was performed using OriginPro software (Origin Lab, 2019, version 9.6.0.172). A one-way ANOVA with a confidence level of 95% (α = 0.05), followed by a Tukey test, was chosen to compare the different quantification methods. The asterisk (*) denotes significance at *p* ≤ 0.05.

## 3. Results and Discussion

### 3.1. Protein Quantification Methods

As non-specific protein quantification methods, the BCA, CB, and Abs_280_ methods were chosen.

#### 3.1.1. BCA Assay

**Figure 1:**
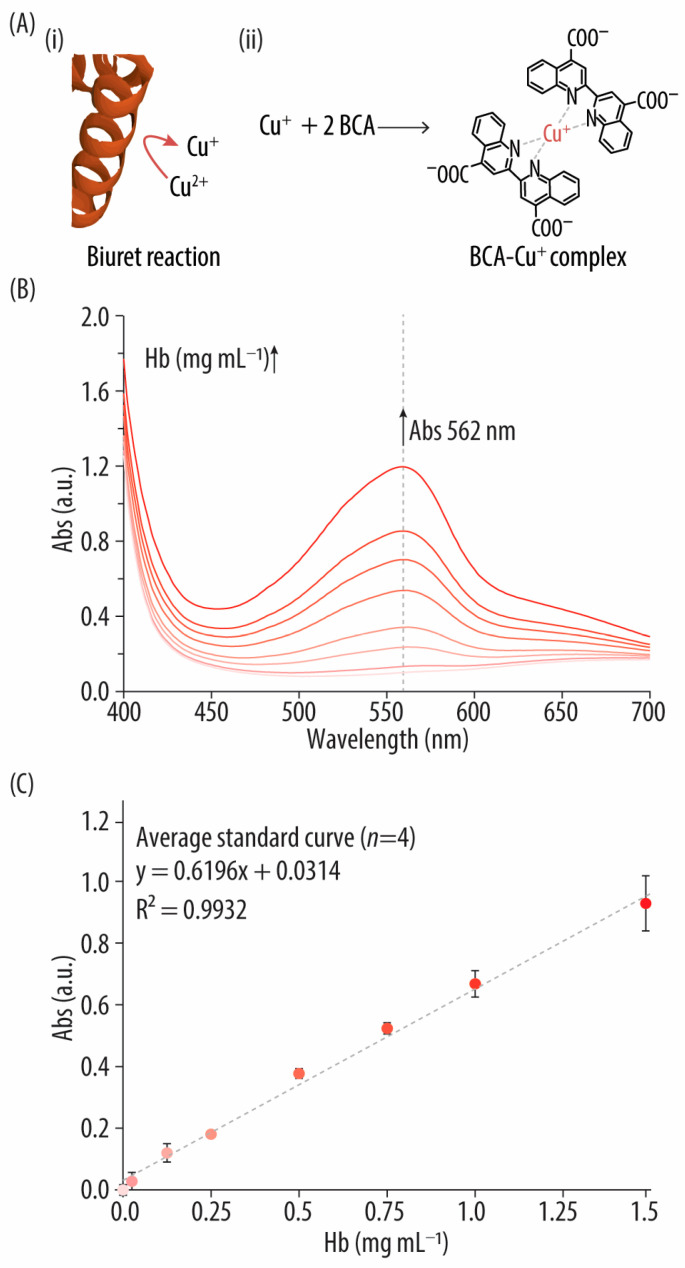
(**A**) Reaction mechanism of the bicinchoninic acid (BCA) assay: (**i**) schematic of the biuret reaction showing the protein’s ability to reduce the cupric ion (Cu^2+^) to the cuprous one (Cu^+^) and (**ii**) formation of the BCA-Cu^+^ complex, which has the characteristic absorbance (Abs) signal at 562 nm. (**B**) UV-vis spectra of the bovine hemoglobin (Hb) standards after incubation with the BCA reagents. (**C**) Standard curve correlating the concentrations of the Hb standards (i.e., 0–1.5 mg mL^−1^) with their Abs signal at 562 nm.

The BCA assay employs a two-step colorimetric process, which involves the chelation of copper by proteins (Figure 1A). In alkaline conditions, proteins through their macromolecular structure, peptide bonds, and certain amino acids (AAs) such as cysteine, its oxidized form cystine, tryptophan, and tyrosine can reduce the cupric ion (Cu^2+^) to the cuprous one (Cu^+^) [26,27]. This reaction, known as the biuret reaction, subsequently leads to the formation of a purple-colored complex when each Cu^+^ is chelated by two BCA molecules forming a stable complex that exhibits a strong Abs signal at 562 nm [22]. This Abs signal is almost linear with protein concentrations in the range 0.02–2 mg mL^−1^ Hb for the microplate procedure [26]. 

Upon mixing BCA reagents (containing Cu^2+^ and BCA in an alkaline medium) with various Hb standards and incubating at 37 °C for 30 min, the UV-vis spectra were examined. Figure 1B shows how the different spectra display the expected Abs peak at 562 nm, characteristic of the BCA-Cu^+^ complex. Higher Hb concentrations correlate with a deeper purple color (Figure S1, Supporting Information) and a stronger BCA-Cu^+^ signal, maintaining linearity up to 1.5 mg mL^−1^ Hb (Figure 1C). It should be noted that free Hb absorbs light at the assay wavelength (i.e., 562 nm), which could potentially lead to interferences. However, at the concentrations used, Hb’s Abs at 562 nm is relatively low compared to the Abs of the BCA-Cu^+^ complex due to its lower extinction coefficient at this wavelength, making its contribution to the overall Abs minimal. Despite being a widely used protein quantification method, limitations of the BCA assay when compared to the other quantification methods studied herein (i.e., CB assay and Abs_280_) include the need for an incubator at 37 °C; longer incubation times; and potential interference from reducing agents, copper chelators (e.g., EDTA), and strong acids or bases (Table 1) [26]. Additionally, the number of peptide bonds, as well as the presence of certain AAs such as cysteine, cystine, tyrosine, and tryptophan may affect accuracy [27]. Therefore, in this study, to avoid over- or underestimation of the protein concentration due to protein-to-protein variability and to minimize interference from Hb’s inherent Abs at 562 nm, the same protein (i.e., bovine Hb) was used to prepare the standards. Nonetheless, BCA offers advantages over the CB assay, such as compatibility with higher concentrations of ionic detergents (up to 5%) [23,26].

#### 3.1.2. Bradford Plus Assay (CB Method)

**Figure 2:**
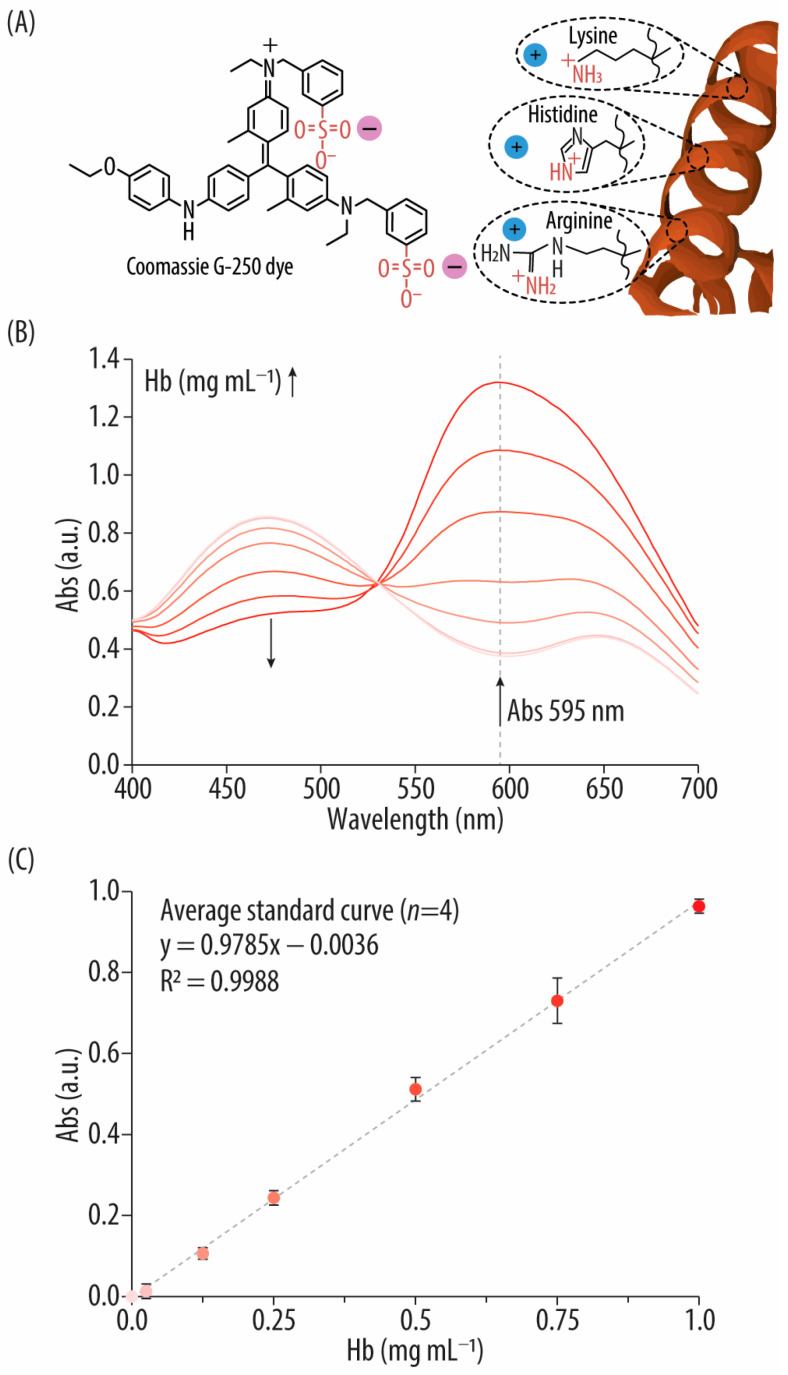
(**A**) Reaction mechanism of the Coomassie blue (CB) assay: electrostatic interactions between the Coomassie G-250 dye and the basic amino acids of a protein. (**B**) UV-vis spectra of the bovine hemoglobin (Hb) standards after incubation with the CB reagent. (**C**) Standard curve correlating the concentrations of the Hb standards (i.e., 0–1 mg mL^−1^) with their absorbance (Abs) signal at 595 nm.

The well-known Bradford assay is a colorimetric procedure based on interactions with the triphenylmethane dye. The specific assay used here is a modification of the traditional Bradford assay with reduced tendency to give non-linear response curves and decreased protein-to-protein variations. The method relies on the interaction of basic AAs (mainly arginine, but also histidine and lysine) with the sulfonic groups of Coomassie G-250 dye in an acidic environment (Figure 2A) [28,29]. This interaction results in a transition from a brown dye that absorbs at 465 nm to a blue product with a characteristic Abs signal at 595 nm (Figure S1, Supporting Information) [23,30]. The Abs is recorded at 595 nm because it is known to present a linear correlation with protein concentration in the 0.1–1.5 mg mL^−1^ Hb range for the microplate procedure [23]. Following incubation of the Hb standards with the Bradford Plus reagent for 10 min, the Abs is recorded [23]. Figure 2B shows the characteristic spectra of the AA-Coomassie G-250 dye complexes which are different depending on the Hb concentration. Specifically, at low Hb concentrations, the Abs spectra display both a maximum at 465 nm and a minimum at 595 nm. Increasing the Hb concentration resulted in a decrease of the intensity of the peak at 465 nm and an increase on the Abs at 595 nm. Figure 2C demonstrates the linear relationship between Hb concentration and Abs at 595 nm in the 0–1 mg mL^−1^ Hb range. Compared to the BCA assay, the CB method offers simpler preparation (i.e., there is no need to prepare a working reagent) and shorter incubation times (i.e., 10 min) that can be conducted at room temperature. However, similarly to the BCA assay, the signal intensity at 595 nm is influenced by the quantity of basic AAs, and thus, protein composition can skew results [31]. Specifically, the number of Coomassie dye ligands bound to each protein is proportional to the number of positive charges found in the protein. This study mitigates such concerns by using the same protein (i.e., bovine Hb) to prepare the standards.

#### 3.1.3. Abs_280_

**Figure 3:**
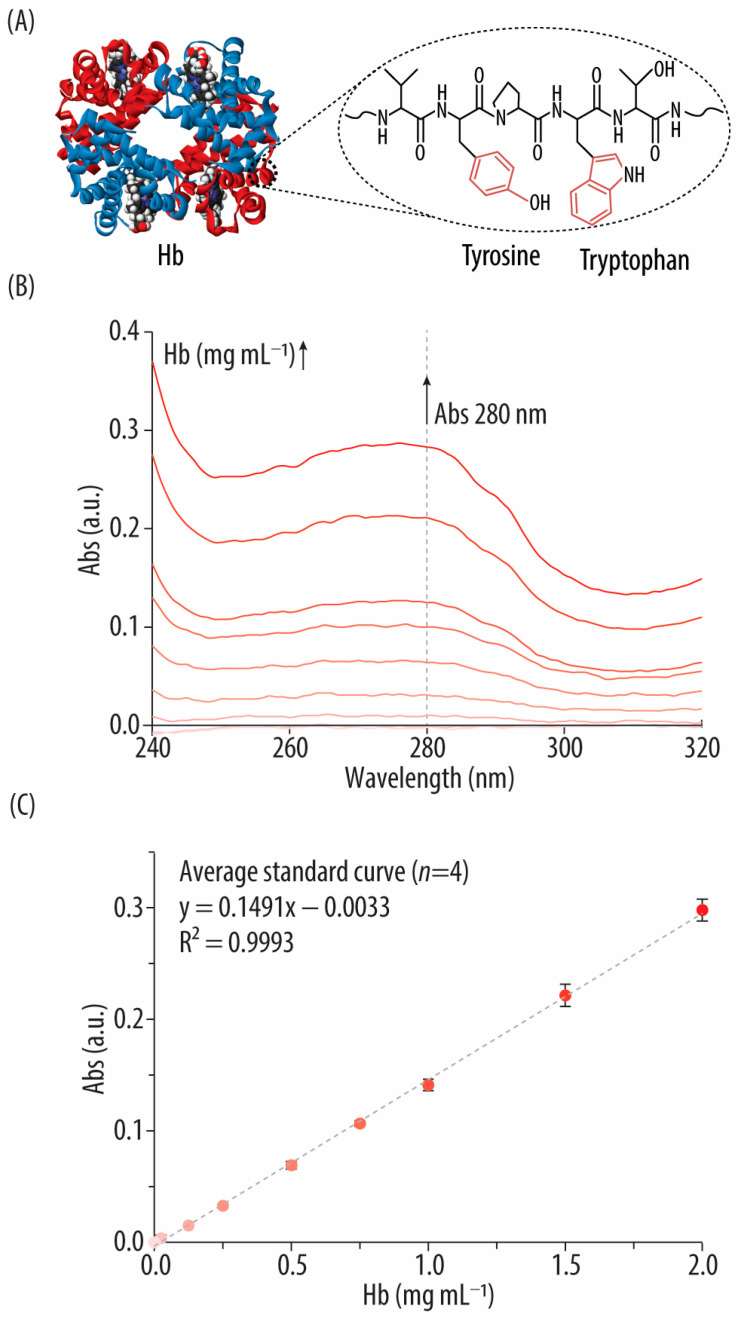
(**A**) Fragment of the hemoglobin (Hb) beta chain amino acid (AA) sequence (i.e., residues 34–38, VYPWT) containing two aromatic AAs that contribute to the absorbance (Abs) signal at 280 nm. (**B**) UV-vis spectra of bovine hemoglobin (Hb) standards in the 240–320 nm range. (**C**) Standard curve correlating the concentrations of the Hb standards (i.e., 0–2 mg mL^−1^) with their Abs signal at 280 nm.

Measuring the Abs_280_ is a traditional, direct method for protein quantification. Some aromatic AAs present in proteins (mainly tryptophan and tyrosine) contain conjugated double bonds that absorb UV light at 280 nm [32]. Since Hb contains six tryptophan and ten tyrosine residues, this method is expected to be suitable for its quantification (Figure 3A) [33,34]. The correlation between Abs_280_ and protein concentration is linear following the Beer-Lambert law. Figure 3B shows the characteristic UV-vis spectra of different Hb standards where the expected increase in the Abs_280_ is observed upon increasing the Hb concentration yielding a linear relationship in the 0–2 mg mL^−1^ Hb range (Figure 3C). 

Since no reagents or incubation are required, the Abs_280_ method is highly reproducible as well as simple, fast, affordable, and convenient. In addition, the Abs_280_ can be measured using a Nanodrop which requires extremely low volumes (i.e., 3 µL for each measurement). Nonetheless, as with the other methods, there are some factors that should be considered. For example, other components that absorb UV light close to 280 nm (e.g., nucleic acids) will interfere with the measurements. Furthermore, the type and number of AAs present and protein structural elements (secondary, tertiary, and quaternary) may slightly alter Abs patterns [35]. Therefore, all factors that may influence protein structure (e.g., pH or ionic strength) should be considered when using this method. In this study, to overcome these limitations, the same protein (i.e., Hb from bovine blood) has been used to prepare the Hb standards, and uniform conditions (i.e., using the same solvents) have been maintained across all measurements.

### 3.2. Quantification Methods Specific for Hb

As Hb-specific quantification methods, the CN-Hb and SLS-Hb assays were chosen.

#### 3.2.1. CN-Hb Method

**Figure 4:**
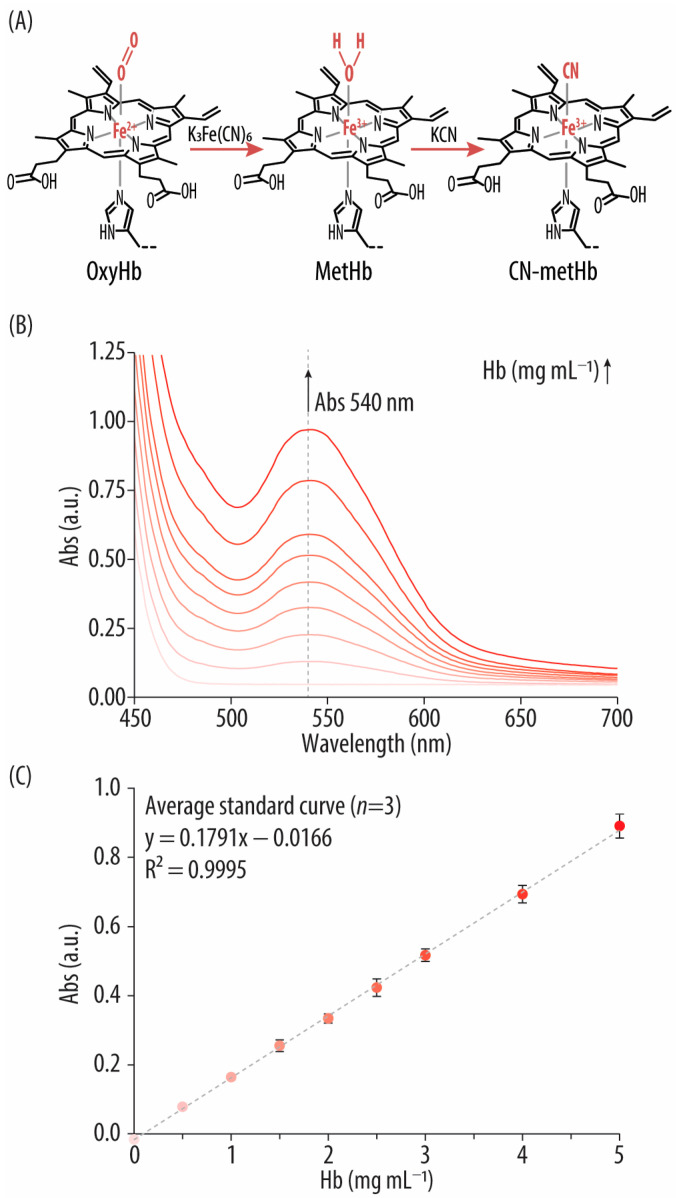
(**A**) Reaction mechanism of the cyan (CN)–hemoglobin (Hb) method: potassium hexacyanoferrate (III) (K_3_[Fe(CN)_6_]) oxidizes Hb into methemoglobin (metHb), which is finally converted into CN-metHb by potassium cyanide (KCN). (**B**) UV-vis spectra of the bovine Hb standards after incubation with K_3_[Fe(CN)_6_] and KCN. (**C**) Standard curve correlating the concentrations of the Hb standard solutions (i.e., 0–5 mg mL^−1^) with their absorbance (Abs) signal at 540 nm.

The hemiglobincyanide (HiCN) method, endorsed by the International Committee for Standardization in Hematology as the reference method for hemoglobinometry in human blood, offers significant advantages, primarily due to the availability of an internationally standardized reference solution [36]. This standard is periodically validated and facilitates the comparison of data all around the world. The basis of the method involves converting Hb into CN-metHb, which is a very stable compound. To do so, blood or an Hb-containing solution is mixed with Drabkin’s reagent (composed of K_3_[Fe(CN)_6_] and KCN). K_3_[Fe(CN)_6_] oxidizes Hb into metHb, which then reacts with KCN forming a stable-colored CN-metHb complex with a characteristic Abs signal at 540 nm (Figure 4A) [36,37]. Then, the Abs at 540 nm is measured and compared with the Hb standards.

In this study, a cyanide-based method that also involves the formation of CN-metHb was conducted following a reported protocol with some modifications [24]. To enable comparison with the SLS-Hb method, the same bovine Hb standards were employed instead of using the international standard HiCN reference solution. Furthermore, instead of using Drabkin’s reagent, K_3_[Fe(CN)_6_] and KCN solutions in TRIS buffer (pH = 7.4) were utilized. Specifically, first, K_3_[Fe(CN)_6_] and then KCN were added to various Hb standards. After 10 min of incubation, the UV-vis spectra showed the characteristic CN-metHb peak at 540 nm (Figure 4B). As expected, the Abs at 540 nm is higher with increasing concentrations of bovine Hb. The linear correlation between Hb concentration and the Abs peak at 540 nm in the 0–5 mg mL^−1^ Hb range is also represented (Figure 4C). The CN-Hb method can measure all forms of Hb except sulfhemoglobin, which is an abnormal irreversible Hb complex that is unable to bind oxygen and is produced by the action of some drugs (e.g., sulfonamides) [38].

#### 3.2.2. SLS-Hb Method

**Figure 5:**
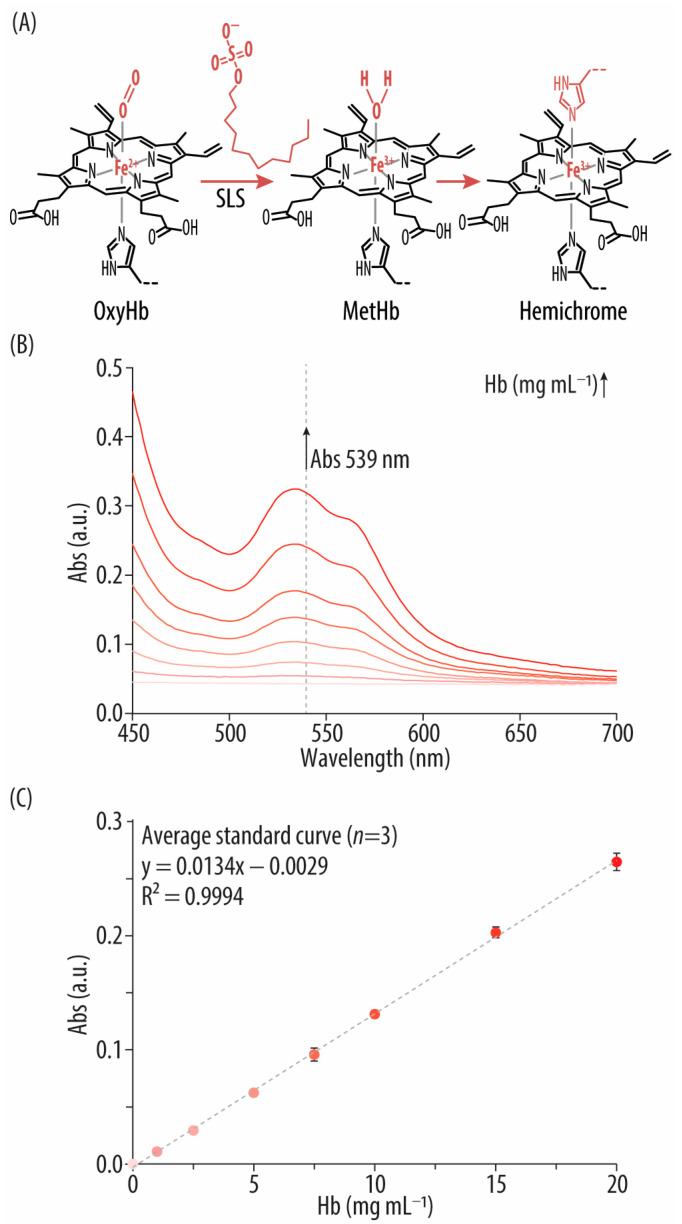
(**A**) Reaction mechanism of the sodium lauryl sulfate (SLS) assay: following SLS addition, hemoglobin (Hb) is transformed into methemoglobin (metHb) rendering a hemichrome-like complex. (**B**) UV-vis spectra of the bovine hemoglobin (Hb) standards after incubation with SLS. (**C**) Standard curve correlating the concentrations of the Hb standards (i.e., 0–20 mg mL^−1^) with their absorbance (Abs) signal at 539 nm.

SLS, also known as sodium dodecyl sulfate, is an anionic surfactant and alkyl sulfonate with oxidative action known for its ability to lyse RBCs and bind to the released Hb [39,40]. This method relies on the oxidation of Hb to metHb, followed by the interaction of SLS with the heme group to form a stable, colored complex detectable via UV-vis spectroscopy (Figure 5A) [41]. The SLS head group can bind electrostatically to the positively charged peptide chains of Hb, while its hydrophobic tail interacts with the heme pocket, forming SLS–hemichrome-like complexes (i.e., SLS-Hb) [25,42]. SLS-Hb exhibits a maximum Abs signal at 534–539 nm [25,43,44]. There is a linear correlation between the Abs at 539 nm from SLS-Hb and the Hb concentration when using an SLS concentration of 1.38 to 3.50 mM in a specific Hb concentration range (i.e., 0–25 mg mL^−1^) [25]. Therefore, for this study, a 2 mM solution of SLS was added to the Hb standards, and their UV-vis spectra were examined. Figure 5B shows the characteristic Abs spectra of SLS-Hb, with a maximum peak at 534 nm, and peak intensity increasing alongside Hb concentration. The linear correlation between Hb concentration and Abs at 539 nm within the 0–20 mg mL^−1^ range is verified (Figure 5C). While both CN-Hb and SLS-Hb methods quickly form their respective complexes, the SLS-Hb method offers distinct advantages over CN-Hb regarding safety and environmental impact, given the toxic and polluting nature of cyanides (Table 1) [25]. Furthermore, the reaction between Hb and SLS is unaffected by variations in pH and temperature, enhancing its practical utility and reliability [25]. However, it should be considered that the range of Hb concentrations used for the SLS-Hb method (i.e., 0–20 mg mL^−1^) is higher than those used in the other techniques, which could make this approach less ideal for detecting low Hb concentrations.

### 3.3. Comparison of the Abs-Based Protein and Specific Hb Quantification Methods 

When comparing the different coefficients of determination (R^2^), both the protein quantification methods (i.e., BCA, CB, and Abs_280_) and the Hb-specific quantification methods (i.e., CN-Hb and SLS-Hb) display similar values. Specifically, the BCA, CB, and Abs_280_ method recorded values of 0.9932, 0.9988, and 0.9993, respectively, while the CN-Hb and the SLS-Hb method recorded values of 0.9995 and 0.9994. The R^2^ values obtained using the Hb quantification methods and the Abs_280_ method are slightly higher than those obtained using the BCA and CB assays. A higher R^2^ indicates a better fit of the data within the respective working ranges to the regression model, compared to other methods.

**Figure 6:**
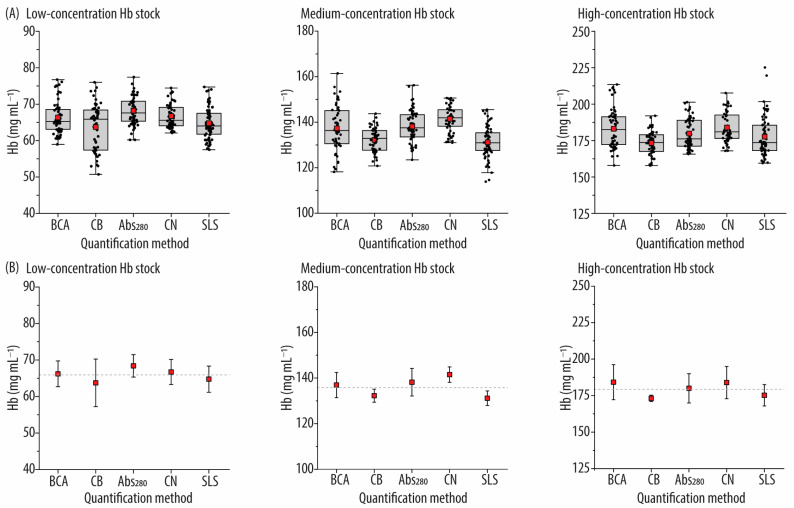
Concentrations of the three hemoglobin (Hb) stocks (low, medium, and high Hb concentrations) assessed by the different assays. (**A**) Boxplots with data points, showing the quartile distribution and the mean value (red square). (**B**) Mean values with standard deviation for *n* = 3 data analysis. The dotted gray lines represent the average Hb concentration of each stock, calculated from the results of all the methods (i.e., 66.0 ± 1.8 mg mL^−1^, 136.0 ± 4.3 mg mL^−1^, and 179.3 ± 5.0 mg mL^−1^ for the low-, medium-, and high-concentration Hb stocks, respectively). BCA: bicinchoninic acid assay; CB: Coomassie blue assay; Abs_280_: absorbance at 280 nm method; CN: cyan–Hb method; SLS: sodium lauryl sulfate–Hb method.

In Figure 6, the concentrations determined for three different Hb stocks (low-, medium-, and high-concentration Hb) using the five assays are presented. Figure 6A shows boxplots of all data points collected, showing the quartile distribution and the mean value indicated with a red square. Figure 6B highlights mean values alongside the standard deviations derived from three independent studies. One key observation is that, regardless of the stock concentration, the Hb concentrations derived from general protein quantification methods are comparable to those obtained from the Hb-specific methods. For instance, in the stock with the lowest Hb concentration, the average Hb concentration determined by the protein quantification methods is 66.1 ± 2.3 mg mL^−1^, whereas it is 65.7 ± 1.4 mg mL^−1^ using the Hb-specific methods. Similar patterns are observed for the medium- and high-concentration Hb stocks (additional details on the average concentrations for each stock through various methods can be found in Table S9, Supporting Information). This consistency suggests that the Hb extraction process has been effective, with no significant contamination from other proteins in the Hb stocks. It is also noted that the standard deviations for the concentrations in the high-concentration Hb stock are larger than those for the low- and medium-concentration Hb stocks across all methods, except for the CB method (Figure 6B). This is likely due to greater dilutions required for the high-concentration Hb stock to facilitate the quantification experiments, which may contribute to higher variability. Interestingly, among the three Hb stocks, the CB and SLS methods consistently report the lowest Hb concentrations. For example, for the high-concentration Hb stock, the CB and SLS-Hb methods yield concentrations of 173.1 ± 2.2 and 175.2 ± 7.3 mg mL^−1^, respectively, while the BCA, Abs_280_, and CN-Hb methods yield concentrations of 184.2 ± 12.0, 180.0 ± 10.0, and 183.9 ± 11.0 mg mL^−1^, respectively.

## 4. Conclusions

In summary, each method examined in this study has its own set of advantages and disadvantages. After thorough comparison and observation of the results across different approaches, it is evident that all methods are effectively suitable for Hb quantification. Nevertheless, the SLS method emerges as the preferred choice due to its specificity for Hb, ease of performance, cost-effectiveness, and safer profile compared to CN-based methods. Furthermore, the SLS-Hb method demonstrates high accuracy, as evidenced by its R^2^ value of 0.9994, reflecting a high degree of linearity. The small standard deviations highlight the method’s precision, indicating consistent and reliable performance across multiple tests. However, it should be noted that higher concentrations of Hb are required to perform the SLS-Hb method, which could be a limitation when there is a need to detect low Hb concentrations.

For scenarios requiring the comparison of the concentration of the same protein across samples—where the absence of other proteins or substances that absorb at 280 nm is ensured—Abs_280_ stands out as the optimal method. Its simplicity, convenience, and minimal volume requirements for samples make it particularly advantageous, especially when all samples are in the same buffer, share the same pH, and have consistent structural properties.

However, the selection of an appropriate method for characterizing HBOCs largely depends on the composition of the carrier. Abs_280_ typically proves unsuitable for HBOCs composed of multiple components, as these components often have Abs in the same spectral region. Here, the SLS-Hb method could offer a superior alternative, although it may occasionally require disassembly of the HBOC prior to quantification. Disassembly of the carrier, often followed by purification, is a common practice to release Hb and facilitate its quantification [10,45]. This process can involve chemical agents to break down complex structures or physical methods such as sonication. The need for disassembly is not unique to the SLS-Hb method; it can also be required for other quantification techniques (e.g., the BCA or the CB assay), especially when dealing with complex or multi-component HBOCs. 

When using UV-vis spectroscopy methods, it is crucial to analyze the Abs spectrum of the HBOC to accurately select an appropriate quantification method. This initial analysis helps identify overlapping absorptions and determine the need for disassembly. Furthermore, the components of the sample and the properties of the formulation, such as pH and the presence of detergents, must be considered to ensure that there are no interferences with the chosen method. This careful approach will facilitate the selection of the most effective and accurate method for protein quantification under varying experimental conditions.

## Figures and Tables

**Table 1:** Summary of the advantages and disadvantages of the different protein quantification methods (bicinchoninic acid (BCA) assay, Coomassie blue (CB) assay, and absorbance at 280 nm (Abs_280_)), and the Hb-specific quantification methods (sodium lauryl sulfate (SLS)–Hb and cyan (CN)–Hb).

Quantification Method	Advantages	Disadvantages
BCA assay	Compatible with samples containing up to 5% ionic detergents	Incubation time (minimum 30 min)
		37 °C Multiple reagents Incompatible with reducing agents or copper-interacting reagents (e.g., EDTA)
CB assay	Compatible with most salts, solvents, buffers, thiols, reducing substances, and chelating agents	Basic conditions and detergents (e.g., SDS) may interfere with the results
	Room temperature	
Abs_280_ method	Direct method	Dependent on protein residues
	No incubation times required, quick	Components that absorb at 280 nm will interfere with the results
	No additional reagents required, convenient	
	Easy Low volume of sample required	
SLS-Hb method	Hb quantification method	
	Cyanide-free method, less toxic Safer to work with than the CN-Hb method	
CN-Hb method	Hb quantification method	Toxic/harmful
		Environmental pollution Multiple reagents

## Data Availability

Data will be made available on request.

## Supplementary Materials

The following supporting information can be downloaded at: https://www.mdpi.com/article/10.3390/biom14091046/s1, Tables S1–S4: dilution ranges for the three different Hb stocks and standard solutions for the protein quantification methods; Tables S5–S8: dilution ranges for the three different Hb stocks and standard solutions for the Hb quantification methods; Figure S1: photographic images of all assays and methods used; Table S9: comparison of the average Hb concentrations of the three different Hb stocks calculated using various protein and Hb quantification methods.
