# Supplementary material for: Comparative Evaluation of UV-Vis Spectroscopy-Based Approaches for Hemoglobin Quantification: Method Selection and Practical Insights
Source: Biomolecules. 2024 Aug 23;14(9):1046. doi: 10.3390/biom14091046 (PMC11430504; doi:10.3390/biom14091046)
Supplement: Supplementary file 1 [file biomolecules-14-01046-s001.zip › biomolecules-3136208-supplementary-re.pdf]

Supporting Information

Comparative Evaluation of UV-Vis Spectroscopy-Based Approaches for Hemoglobin Quantification: Method Selection and Practical Insights

Clara Coll-Satue *, Michelle Maria Theresia Jansman and Leticia Hosta-Rigau *

Center for Nanomedicine and Theranostics, Department of Health Technology, Technical University of Denmark, Nils Koppels Allé, Building 423, 2800 Kongens Lyngby, Denmark; michja@dtu.dk

* Correspondence: clcosa@dtu.dk (C.C.-S.); leri@dtu.dk (L.H.-R.); Tel.: +45-4525-8155 (L.H.-R.)

Specific dilutions prepared for the protein quantification methods

1) Low-concentration Hb stock

Dilution	Hb (µL)	MQ (µL)
x25	40 (stock)	950
x50	400 (x25)	400
x75	200 (x25)	400
x100	400 (x50)	400
x150	100 (x25)	500
x200	75 (x25)	525
x300	100 (x100)	200
x400	200 (x200)	200

2) Medium-concentration Hb stock

Dilution	Hb (µL)	MQ (µL)
x25	40 (stock)	950
x75	200 (x25)	400
x100	150 (x25)	450
x150	100 (x25)	500
x200	75 (x25)	525
x250	60 (x25)	540
x300	100 (x100)	200
x400	200 (x200)	200
x600	200 (x300)	200

3) High-concentration Hb stock

Dilution	Hb (µL)	MQ (µL)
x25	40 (stock)	950
x100	150 (x25)	450
x150	100 (x25)	500
x200	75 (x25)	525
x250	60 (x25)	540
x300	100 (x100)	200
x400	200 (x200)	200
x500	200 (x250)	200
x700	200 (x400)	150

4) Standard line

ID	Hb(mg mL^{-1})	Hb (µL)	MQ (µL)
A	2	300 (stock)	0
B	1.5	375 (stock)	125
C	1	325 (stock)	325
D	0.75	175 (B)	175
E	0.5	325 (C)	325
F	0.25	325(E)	325
G	0.125	325 (F)	325
H	0.025	100 (G)	400
I	0	0	400

Tables S1–S4. Dilution ranges for the three different hemoglobin (Hb) stocks (low-, medium-, and high-concentration) and standard solutions for the protein quantification methods. The standard line was prepared as described in the Pierce^{TM} BCA assay protocol. In blue, the dilutions used in the bicinchoninic acid (BCA) assay. Boxed in brown, the dilutions used in the Coomassie blue (CB) assay. All dilutions were measured for absorbance at 280 nm (Abs_{280}).

Specific dilutions prepared for the Hb quantification methods

5) Low-concentration Hb stock

Dilution	Hb (µL)	TRIS (µL)
x4	25 (stock)	75
x5	180 (stock)	720
x10	450 (x5)	450
x15	300 (x5)	600
x20	200 (x10)	200
x25	520 (x10)	780
x30	300 (x15)	300
x40	300 (x20)	300
x50	650 (x25)	650
x75	400 (x50)	200
x100	300 (x50)	300

6) Medium-concentration Hb stock

Dilution	Hb (µL)	TRIS (µL)
x8	12.5 (stock)	87.5
x10	80 (stock)	720
x20	450 (x10)	450
x30	300 (x10)	600
x40	300 (x20)	300
x50	520 (x20)	780
x60	300 (x30)	300
x75	100 (x50)	50
x100	600 (x50)	600
x150	600 (x100)	300
x300	300 (x150)	300

7) High-concentration Hb stock

Dilution	Hb (µL)	TRIS (µL)
x10	70 (stock)	630
x15	20 (stock)	280
x20	500 (x10)	500
x30	200 (x15)	200
x40	450 (x20)	450
x50	480 (x20)	720
x60	300 (x30)	300
x80	300 (x40)	300
x100	600 (x50)	600
x150	600 (x100)	300
x300	300 (x100)	300

8) Standard line

ID	Hb (mg mL^{-1})	Hb (µL)	TRIS (µL)
A	20	850 (stock)	-
B	15	75 (A)	25
C	10	750 (A)	750
D	7.5	525 (C)	175
E	5	450 (C)	450
F	4	480 (D)	420
G	3	270 (C)	630
H	2.5	300 (E)	300
I	2	550 (G)	550
J	1.5	300 (H)	300
K	1	450 (J)	450
L	0.5	300 (L)	300
M	0	0	600

Table S5–S8. Dilution ranges for the three different hemoglobin (Hb) stocks (low-, medium-, and high-concentration) and standard solutions for the Hb quantification methods. In blue, the dilutions used in the sodium lauryl sulfate (SLS)-Hb method. Boxed in brown, the dilutions used in the cyano (CN)-Hb method.

Appearance of the various methods used to quantify Hb

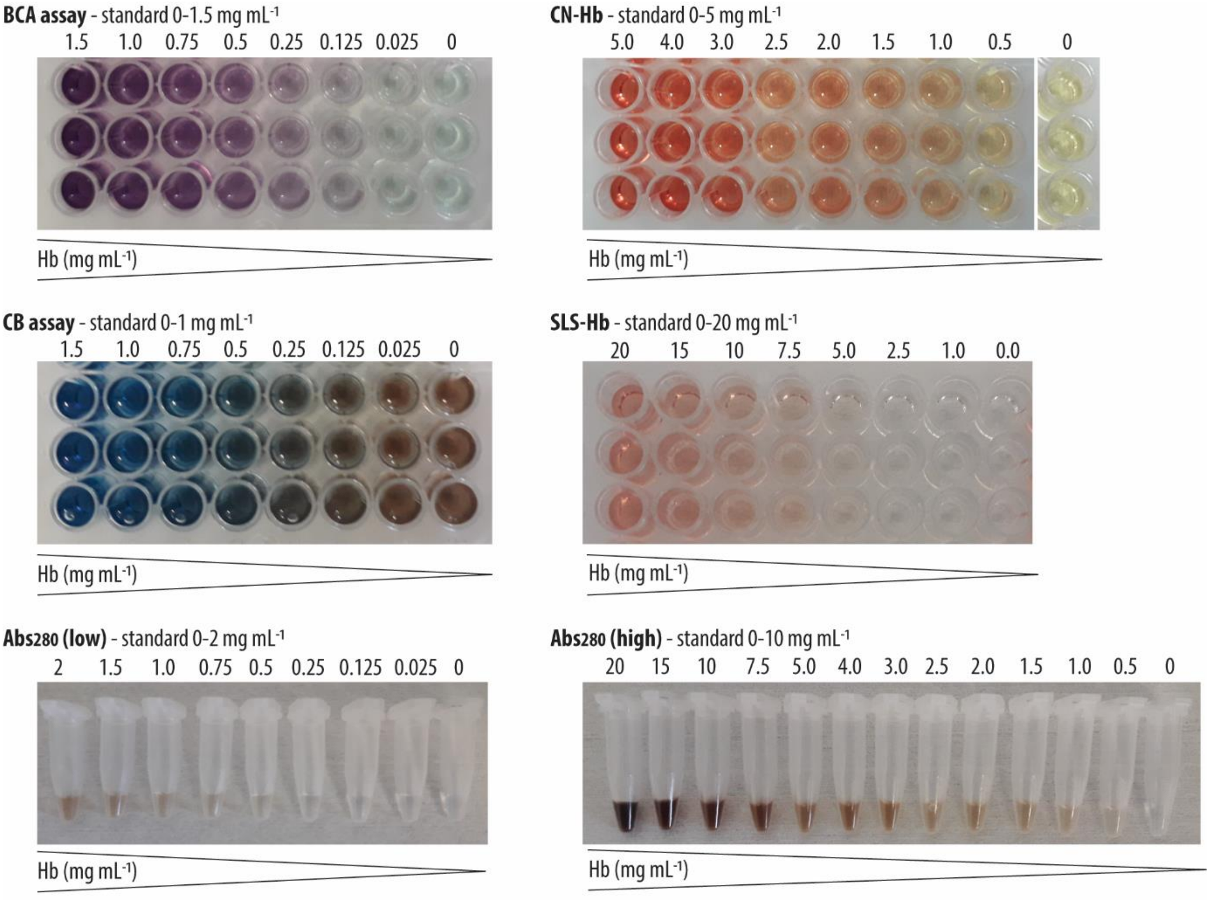

Figure S1. Photographic images of all assays and methods used. The top four images show the technical triplicates of standard dilutions obtained in the different 96-well plate assays. The bottom two images show tubes with the standard dilutions used to measure the absorbance at 280 nm (Abs_{280}). BCA: bicinchoninic acid, CB: Coomassie Blue, CN-Hb: cyano-Hb, SLS: sodium lauryl sulfate.

Summary of Hb concentrations obtained with the various quantification methods

	Protein quantification methods				Hb quantification methods		
**Hb concentration stock**	**BCA**	**CB**	**Abs_{280}**	**Average**	**CN-Hb**	**SLS-Hb**	**Average**
**Low**	66.2 ± 3.5	63.7 ± 6.5	68.4 ± 3.1	66.1 ± 2.3	66.7 ± 3.4	64.8 ± 3.6	65.7 ± 1.4
**Medium**	136.9 ± 5.5	132.3 ± 2.9	138.1 ± 6.0	135.8 ± 3.1	141.5 ± 3.4	131.1 ± 3.2	136.3 ± 7.3
**High**	184.2 ± 12.0	173.1 ± 2.2	180.0 ± 10.0	179.1 ± 5.6	183.9 ± 11.0	175.2 ± 7.3	179.6 ± 6.1

Table S9. Comparison of the average hemoglobin (Hb) concentration of the three different Hb stocks (low-, medium-, and high-concentration) calculated using various protein and Hb quantification methods. BCA: bicinchoninic acid, CB: Coomassie Blue, Abs: absorbance, CN-Hb: cyano-Hb, SLS: sodium lauryl sulfate.
